# The relationship between corruption and chronic diseases: evidence from Europeans aged 50 years and older

**DOI:** 10.1007/s00038-020-01347-w

**Published:** 2020-03-26

**Authors:** Lorenzo Ferrari, Francesco Salustri

**Affiliations:** 1 Department of Economics and Finance, University of Rome “Tor Vergata”, Rome, Italy; 2 John Cabot University, Rome, Italy; 3 Health Economics Research Centre, Nuffield Department of Population Health, University of Oxford, Richard Doll Building, Old Road Campus, Oxford, OX3 7LF UK

**Keywords:** Corruption, Public health, Chronic diseases, Europe

## Abstract

**Objectives:**

Do people living in more corrupted countries report worse health? We answer this question by investigating the relationship between country-level corruption and the number of chronic diseases for a sample of Europeans aged above 50.

**Methods:**

We link a rich panel dataset on individual health and socio-demographic characteristics with two country-level corruption indices, analyse the overall relationship with pooled ordinary least squares and fixed-effect models, explore heterogeneous effects driven by country and individual factors, and disentangle the effect across different public sectors.

**Results:**

Individuals living in more corrupted countries suffer from a higher number of chronic diseases. The heterogeneity analysis shows that (1) health outcomes are worsened especially for respondents living in relatively low-income countries; (2) the health of females and people with poor socio-economic status is more affected by corruption; (3) the corruption–health negative link mainly occurs for cardiovascular diseases and ulcers; (4) only corrupted sectors linked with healthcare are associated with poorer health.

**Conclusions:**

We inform the policy debate with novel results in establishing a nexus between corruption and morbidity indicators.

**Electronic supplementary material:**

The online version of this article (10.1007/s00038-020-01347-w) contains supplementary material, which is available to authorized users.

## Introduction

An efficient provision of healthcare services is crucial to individual and social well-being in both developing and developed countries. In developed countries, for instance, healthcare represents one of the largest government expenditures [6% of Gross Domestic Product (GDP) in countries belonging to the Organisation for Economic Co-Operation and Development (OECD), Joumard et al. (2010)]. At the same time, several elements have been identified, suggesting that this sector is particularly prone to corruption (European Commission 2013; Petkov and Cohen 2016; Hussmann 2011).

A longstanding and most popular definition understands corruption as “the abuse of power by a public official for private gain” (Miller 2018). As such, it is not clear a priori whether corruption threatens the health of citizens—for instance, through a reduction in the quality, the accessibility, or the trust of healthcare—or may serve as a channel for the informal economy to increase the quantity of healthcare. In general, while the effects of corruption on economic and social outcomes have long been debated, scholars have not reached yet an uncontroversial conclusion, as the sign of the effect crucially depends on the questions addressed.

A large body of literature acknowledges the detrimental role of corruption on several socio-economic indicators, such as economic growth (Mo 2001; Ehrlich and Lui 1999, among others), income inequality (Gupta et al. 2002), investments (Campos et al. 1999), productivity (Lambsdorff 2003), social trust (Li and Wu 2010; Richey 2010), and trust in institutions (Torcal and Christmann 2018). Empirically, various authors (Mauro 1998; Gupta et al. 2002) show that higher level of corruption is associated, in general, with an alteration of the government spending composition and, in particular, to a reduction in the ratio of healthcare and education spending to GDP. On the link between corruption and health outcomes, most papers find negative relations employing aggregate health outcomes, such as mortality rates and life expectancy at birth (Gupta et al. 2000; Muldoon et al. 2011; Nadpara and Samanta 2015). Interestingly, many of these findings document a stronger effect in developing countries, where corruption tends to be endemic, and widespread income inequalities are more likely to imply the exclusion of a considerable portion of the population from the healthcare system. Another channel of the detrimental effect of corruption on health is the effectiveness of public health expenditure (Rajkumar and Swaroop 2007; Factor and Kang 2015, among others).

On the contrary, few but still non-negligible papers claim that a certain degree of ‘efficient corruption’ may lead to higher economic growth (Leff 1964; Huntington 2006), especially in countries characterised by weak or inefficient institutions (Méon and Weill 2010). In particular, bribery actions may help firms in overcoming bureaucracy-related stalls and consequently speed-up productivity. This view claims that “if the government erred in its decision, the course made possible by corruption may well be the better one” (Leff 1964); corruption would thus act as a ‘greasing’ mechanism capable of speeding up the inefficient wheels of bureaucracy.

Our research also speaks to the health economics literature that deals with health outcomes. This literature mostly relies on aggregate mortality indicators, such as life expectancy at birth, infant and mother mortality rates, and children immunisation rate (Gupta et al. 2002; Muldoon et al. 2011; Dincer and Teoman 2019). However, morbidity indicators would be preferable as they account for health gains due to specific treatments (Joumard et al. 2010; Anderson and Horvath 2004). In addition, the use of individual-level data would provide more insights as it allows capturing heterogeneous effects depending on socio-demographic characteristics. In this respect, Bollyky et al. (2019) have recently shown the negative link between democracy and cause-specific mortality.

In this paper, we explore the relationship between corruption and individual morbidity indicators. To this purpose, we match data on country-level corruption, as measured by the Corruption Perception Index (hereinafter, CPI), with longitudinal data on individual health outcomes for a panel of European citizens aged 50+. In our estimations, we employ both pooled ordinary least squares (OLS) and fixed-effects empirical methodologies.

The contribution of our paper to the existing literature is threefold. First, there are no studies that investigate the link between corruption and health outcomes in high-income countries. The literature has mainly focussed on aggregate outcomes in developing countries, and we have surprisingly not found any papers assessing the link between corruption and morbidity outcomes in the European Union. Second, we rely on morbidity rather than mortality indicators as our dependent variable. This represents an innovation since the literature has traditionally focussed on indicators such as life expectancy at birth and child mortality. We believe that our morbidity indicator (i.e. the number of chronic diseases) represents a much better proxy for the healthcare quality in high-income countries, where mortality indicators tend to be similar and chronic diseases are becoming more widespread (Seo et al. 2017; Becchetti et al. 2017). Third, differently from most of the existing literature, our dataset makes it possible to observe the variability of selected morbidity indicators at individual level. This allows us to explore the heterogeneous effect of corruption across socio-demographic characteristics. Analogously, data on the occurrence of specific chronic diseases allow us to investigate the heterogeneous relationship between corruption and the probability of being affected by each disease.

### Transmission channels of corruption in healthcare

The existing literature identifies six different types of healthcare corruption potentially arising from five kinds of interactions between service providers, payers, customers of healthcare services, and the medical industry (European Commission 2013; Bruckner 2019). Bribery typically enters the relation between providers and patients and consists in the payment of a bribe in exchange for the access to the service. This practice seems to be particularly widespread in countries where healthcare services are financed through general taxation and providers’ salaries are relatively low. Informal payments have been generally associated with healthcare systems in former socialist countries, but some evidence suggests that they are also widespread in some Mediterranean countries like Greece and Italy. Corruption in the procurement of pharmaceuticals and medical devices, together with improper marketing relations, characterises the relation between the medical industry and providers. This includes, for instance, the alteration of the lawful bidding process and the retention of providers through small gifts. Improper marketing relations can also arise between the industry and the regulator, when the medical industry may employ its lobbying power to influence policies. The misuse of high-level positions involves all actors but patients and is characterised by high-level relations among industry, regulators, politicians, and providers, benefiting the medical industry. Practices in this group include regulatory capture and conflict of interest or reimburses for services which are not carried out or do not necessary characterise the relation between providers and payers. These practices, which potentially lead to an increase in insurance premia and, consequently, to the exclusion of the poorest from basic healthcare, is particularly widespread in countries where healthcare is financed through public or private insurance coverage. In Europe, this happens especially in Central Europe countries such as Germany, France, and The Netherlands. The last type of corruption involves providers only and concerns the embezzlement of drugs and medical devices illegally sold or used for private gain.

## Methods

### Data

We combine two sources of data, namely the Survey of Health, Ageing, and Retirement in Europe (SHARE) and CPI. SHARE is a longitudinal dataset that contains information on health and socio-economic status of a sample of Europeans aged above 50 (Börsch-Supan et al. 2013). We focus on waves 1, 2, 4, 5, and 6, covering the period 2004–2015 (we exclude wave 3 as it refers to a retrospective survey of people life’s history). We consider the following countries: Austria, Belgium, Czech Republic, Denmark, Estonia, France, Germany, Greece, Italy, The Netherlands, Poland, Slovenia, Spain, and Sweden. CPI is a worldwide-known index provided by Transparency International, a non-governmental non-profit organisation whose purpose is to fight corruption through the proposal of anti-corruption measures. This index ranks 176 countries from the most to the least corrupted on a 0–10 scale and is calculated annually on the basis of a methodology involving surveys and assessments from leading institutions on corruption like the World Bank, the World Economic Forum, and the African Development Bank. The index has been widely used in the literature and its validity has been tested in comparison with many different indicators (Wilhelm 2002). In particular, CPI is highly correlated with two other proxies of corruption, i.e. black market activity and over-abundance of regulation. As a robustness check, we also employ the Control of Corruption index (hereinafter, CoC), provided annually by the World Bank, and different sector-specific corruption perception measures (hereinafter, CP_s_ for each sector *s* as described below) provided biennially by Eurobarometer, as alternative proxies for corruption. CoC ranges from − 2.5 to 2.5 and, similarly to CPI, ranks countries from the least to the most corrupted based on perceived levels of petty and grand corruption (for a comparison between the two indices see Rohwer 2009). CP_s_ measures national perception of corruption in the following sectors *s*: public healthcare, police, judicial courts, national politicians, public tenders, and the concession of building and business permits (see the Electronic Supplementary Material (ESM)-B for details on Eurobarometer).

The merge of these two independent datasets on individual health outcomes and expert assessment of national corruption ensures that our estimates do not suffer from any bias resulting from the same person answering possibly related questions.

### Econometric analysis

To investigate the effect of corruption on our variable of interest, we assume that the health outcome for individual *i* in wave *t* is related to *CPI* in country *c* and wave *t* according to the following econometric specification:

1 $${\text{Health}}_{i,t} = \beta_{0} + \beta_{1} {\text{CPI}}_{{c_{i} ,t}} + \beta_{2} X_{i,t} + \beta_{3} Z_{i,t} + {\text{Region}}_{i} + {\text{Wave}}_{t} + \varepsilon_{i,t} ,$$

where ${\text{Health}}_{i,t}$ represents the number of chronic diseases that respondent *i* declared as diagnosed by a doctor during wave *t* and ${\text{CPI}}_{{c_{i} ,t}}$ is our proxy for the level of corruption in country $c_{i}$ where respondent $i$ resided in wave $t$. Alternatively, we replace ${\text{Health}}_{i,t}$ with the probability of respondent *i* to be affected by at least one chronic disease. We are interested in estimating the coefficient $\beta_{1}$, which captures the effect of ${\text{CPI}}_{{c_{i} ,t}}$ on ${\text{Health}}_{i,t}$.

We further include a wide set of covariates to account for individual characteristics that are likely to be correlated with health outcomes. More specifically, $X_{i,t}$ is a vector of socio-demographic characteristics of individual $i$ during wave $t$ and includes gender, age, education in years, logarithm of household income, current job status (retired, employed, unemployed, permanently disabled, and home-maker), marital status (married, not married, divorced, and widowed), place of living (large city, small city, and rural area), and number of sons and daughters.

The vector ${\text{Z}}_{i,t}$ includes individual characteristics related to healthy lifestyle, i.e. a dummy equal to 1 if the respondent has ever smoked daily, intensity of alcohol consumption (ranging from non-drinker to everyday drinker), degree of participation in sport activities (never, sometimes, often), and body mass index category (underweight, normal weight, overweight, and obese). We further include a full set of regional and wave fixed effects, in order to capture the impact of any unobserved geographic and time trend. Standard errors are clustered at the regional level to allow for autocorrelation of observations located in the same geographical area.

Moreover, we exploit the longitudinal nature of *SHARE* to estimate a fixed-effects specification of (1). Panel methodologies successfully deal with the endogeneity arising from the correlation of time-invariant unobserved covariates and our independent variable, CPI. As a consequence, the use of the fixed-effects estimator provides further robustness and credibility to our analysis.

## Results

### Descriptive statistics and preliminary evidence

Our final dataset includes 161,641 observations (defined as individual-wave pairs), referred to 69,432 individuals, 92% of which took part in two or more waves. Mean age is 66.5 years, 55% of respondents are female, and the average years of education are 11. Most of the observations are either married (69.5%) or widowed (15%) and have, on average, slightly more than two children. More than half of respondents are either retired (58%) or employed (26%). As for lifestyle characteristics, almost 47% of respondents currently smoke or used to smoke daily, and more than 40% are not engaged with any physical activity. Approximately 60% of individuals are either overweight or obese. Chronic diseases are quite widespread in our sample, as more than 60% of the respondents declared to be affected by at least one. Of all the diseases, cardiovascular diseases are the most frequent (91.1%), followed by age-related (14%) and respiratory (9.8%) diseases, cancer (8%), ulcer (7.4%), and hip or femur fracture (2.9%). See ESM-A for further details.

**Fig. 1:**
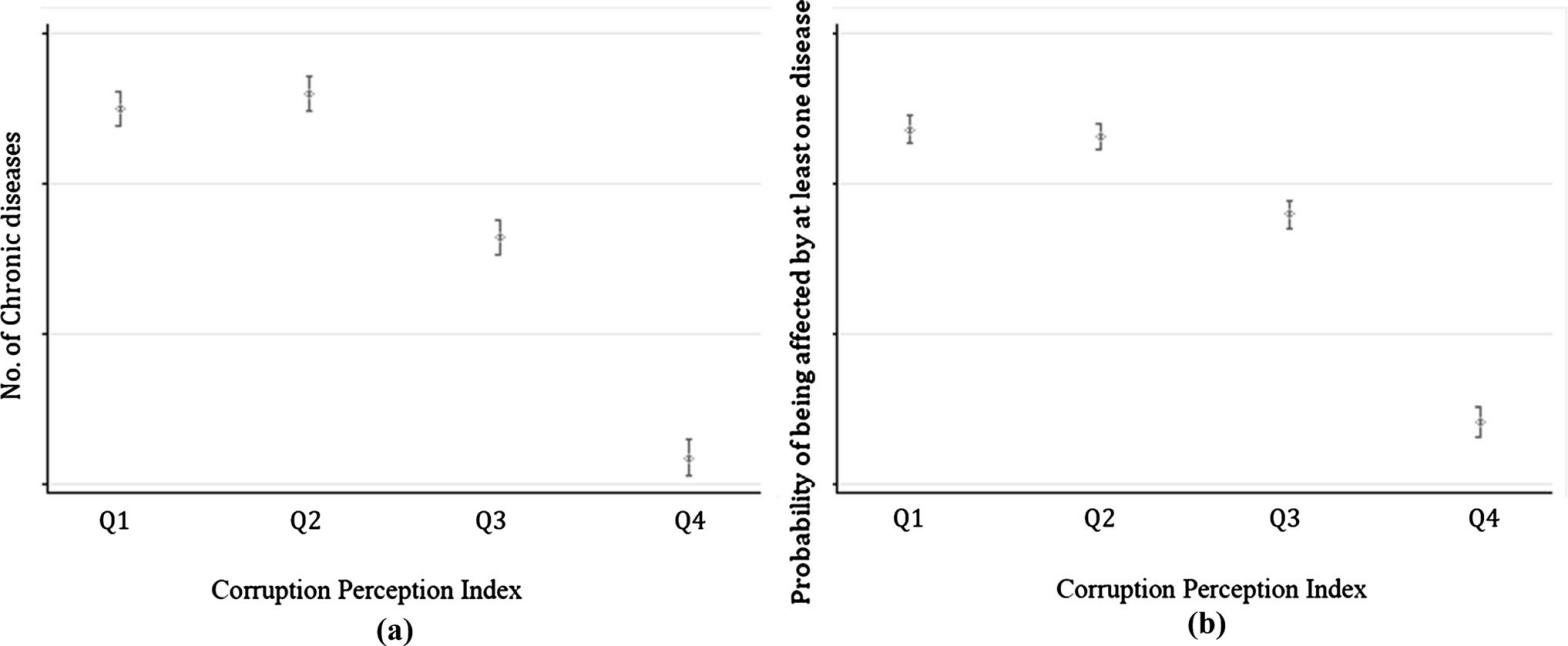
Number and probability of chronic diseases for Corruption Perception Index quartiles. *Notes* Europe, data from 2004 to 2015. Average number of chronic diseases (**a**) and the probability of being affected by at least one chronic diseases (**b**) with 95% confidence intervals, by Corruption Perception Index quartiles. Q1 represents the highest quartile (the least corrupted countries) and Q4 the lowest quartile (the most corrupted countries)

Figure 1 provides descriptive evidence of the effect of CPI on our variables of interest. Even though at a descriptive level, individuals living in less corrupted countries (i.e. lower CPI) display on average more diseases and are more likely to be affected by at least one disease. Moreover, mean values are significantly different for three quartiles. In addition, both the number of chronic diseases and CPI do vary over time and across countries (see ESM-A). These preliminary findings call for a further econometric investigation that we detail below.

### Econometric findings

Pooled OLS results (Table 1, columns 1–4) show that an increase in CPI is significantly associated with a reduction in the individual number of chronic diseases, and estimates are robust to the inclusion of socio-demographic and healthy lifestyle covariates. The existence of a significantly negative correlation is confirmed by fixed-effects estimates (Table 1, columns 5–8). The magnitude of the coefficients is very similar for the two specifications, providing robustness to the results. As for the other covariates, the elders and males are associated with more diseases (ESM-A), as intuitive for the elders and widely documented in the medical literature for gender differences [see, among others, Nkomo et al. (2006) and Booth et al. (2006)]. Health-related behaviours (i.e. smoking, alcohol consumption, and the Body Mass Index) are also positively associated with the number of chronic diseases (ESM-A). Results, in line with the existing literature, support the claim that individuals in relatively more corrupted countries display average worse health outcomes. In particular, a unit increase in CPI is associated with a reduction in the number of chronic diseases of approximately 0.07. The magnitude of the estimated coefficients may look relatively small if we consider that we need a 13-unit increase in CPI for a unit reduction in the number of chronic diseases. A plausible explanation lies in the distribution of the number of chronic diseases in our sample, as almost 70% of respondents are either not affected by morbidity or suffer from one only. The average number of chronic diseases, moreover, is only slightly larger than 1 and does not display a huge variability in absolute terms.

**Table 1:** The impact of Corruption Perception Index on the number of chronic diseases

Dependent variable	(1)	(2)	(3)	(4)	(5)	(6)	(7)	(8)
No. of chronic diseases	OLS	OLS	OLS	OLS	FE	FE	FE	FE
CPI	− 0.0583**	− 0.0582**	− 0.0703***	− 0.0696***	− 0.0682***	− 0.0677***	− 0.0696***	− 0.0691***
	(0.0257)	(0.0254)	(0.0261)	(0.026)	(0.0248)	(0.0249)	(0.0247)	(0.0249)
Age	0.0366***	0.0269***	0.0326***	0.0261***	0.0219	0.0224	0.0243	0.0247
	(0.0009)	(0.0007)	(0.0008)	(0.0008)	(0.0268)	(0.0266)	(0.0261)	(0.0259)
Female	− 0.0298*	− 0.0546**	− 0.0433***	− 0.0514***	–	–	–	–
	(0.0168)	(0.0212)	(0.012)	(0.0145)				
Region FE	Yes	Yes	Yes	Yes	No	No	No	No
Wave FE	Yes	Yes	Yes	Yes	Yes	Yes	Yes	Yes
Socio-demographics	No	Yes	No	Yes	No	Yes	No	Yes
Healthy lifestyle	No	No	Yes	Yes	No	Yes	No	Yes
Mean	1.1789	1.1789	1.1789	1.1789	1.1789	1.1789	1.1789	1.1789
Observations	161,641	161,641	161,641	161,641	161,641	161,641	161,641	161,641
*R*-squared	0.105	0.126	0.165	0.175	0.084	0.093	0.11	0.12

Europe, data from 2004 to 2015. *OLS* ordinary least squares, *FE* fixed effects, *CPI* Corruption Perception Index, *FE* fixed effects. OLS (columns 1–4) and FE (columns 5–8) estimates of specification (1). Region FE include regions in Austria, Belgium, Czech Republic, Denmark, Estonia, France, Germany, Greece, Italy, The Netherlands, Poland, Slovenia, Spain, and Sweden. Wave FE include waves 1, 2, 4, 5, and 6 covering the period 2004–2015. Robust standard errors clustered at regional level in parenthesis

***p < 0:01; **p < 0:05; *p < 0:1

These considerations need a further investigation on whether CPI affects the probability that a respondent reports to suffer from at least one chronic disease in a given wave. To do so, we replace ${\text{Health}}_{i,t}$ in (1) with a dummy variable equal to 1 if the respondent is affected by at least one chronic disease in wave $t$ and estimate both a pooled OLS and a fixed-effects linear probability model. Results are robust to all econometric specifications and show that a unit increase in CPI is significantly associated with a reduction in the probability of being affected by at least one chronic disease by approximately 2% (Table 2). Notice that linear probability models do not account for any nonlinearity in the effect of corruption, and it does not constraint predicted outcomes to be between 0 and 1. To tackle this issue, we also run logit and probit specifications (ESM-A). Interestingly, the effect of CPI is still negative and significant when considering the probability of suffering from a second, a third, and a forth disease (ESM-A). All these findings corroborate our claim that corruption has an overall detrimental effect on health outcomes.

**Table 2:** The impact of Corruption Perception Index on the probability of suffering from at least a chronic disease

Dependent variable	(1)	(2)	(3)	(4)	(5)	(6)	(7)	(8)
At least one chronic diseases	OLS	OLS	OLS	OLS	FE	FE	FE	FE
CPI	− 0.0138**	− 0.0141**	− 0.0179**	− 0.0179**	− 0.0193***	− 0.0193***	− 0.0198***	− 0.0197***
	(0.0069)	(0.0069)	(0.0069)	(0.0069)	(0.0065)	(0.0066)	(0.0065)	(0.0066)
Age	0.0128***	0.0089***	0.0120***	0.0091***	0.0104	0.0105	0.0112	0.0113
		(0.0091)	(0.009)	(0.0089)	(0.0089)			
Female	− 0.0163***	− 0.0241***	− 0.013***	− 0.0165***	–	–	–	–
	(0.0051)	(0.0064)	(0.0045)	(0.0053)				
Region FE	Yes	Yes	Yes	Yes	No	No	No	No
Wave FE	Yes	Yes	Yes	Yes	Yes	Yes	Yes	Yes
Socio-demographics	No	Yes	No	Yes	No	Yes	No	Yes
Healthy lifestyle	No	No	Yes	Yes	No	No	Yes	Yes
Mean	0.6383	0.6383	0.6383	0.6383	0.6383	0.6383	0.6383	0.6383
Observations	161,641	161,641	161,641	161,641	161,641	161,641	161,641	161,641
*R*-squared	0.083	0.098	0.122	0.13	0.072	0.077	0.089	0.093

Europe, data from 2004 to 2015. *OLS* ordinary least squares; *FE* fixed effects; *CPI* Corruption Perception Index; *FE* fixed effects. OLS (columns 1–4) and FE (columns 5–8) estimates of specification (1), with a dummy if affected by at least one disease as dependent variable. Region FE include regions in Austria, Belgium, Czech Republic, Denmark, Estonia, France, Germany, Greece, Italy, The Netherlands, Poland, Slovenia, Spain, and Sweden. Wave FE include waves 1, 2, 4, 5, and 6 covering the period 2004–2015. Robust standard errors clustered at regional level in parenthesis

***p < 0:01; **p < 0:05; *p < 0:1

As an additional robustness check, we run (1) using CoC index as a proxy for country-level corruption (see ESM-A for descriptive statistics on CoC). Results are presented in Table 3 and confirm our baseline findings. Note that the two perception indices are highly correlated (0.98) and this explains the similarity of our results.

**Table 3:** The impact of Control of Corruption on the number and probability of chronic diseases

Dependent variable	No. of chronic diseases		At least one chronic diseases	
	(1)	(2)	(3)	(4)
	OLS	FE	OLS	FE
CoC	− 0.139**	− 0.18***	− 0.04**	− 0.0529***
	(0.054)	(0.0476)	(0.015)	(0.0137)
Age	0.0261***	0.0052	0.091***	0.0057
	(0.0007)	(0.024)	(0.0003)	(0.0081)
Female	− 0.0514***	–	− 0.0165***	–
	(0.0145)		(0.0053)	
Region FE	Yes	No	Yes	No
Wave FE	Yes	Yes	Yes	Yes
Socio-demographics	Yes	Yes	Yes	Yes
Healthy lifestyle	Yes	Yes	Yes	Yes
Mean	1.1789	1.1789	0.6383	0.6383
Observations	161,641	161,641	161,641	161,641
*R*-squared	0.175	0.13	0.068	0.089

Europe, data from 2004 to 2015. *OLS* ordinary least squares, *FE* fixed effects, *Coc* Control of Corruption Indexs. OLS (columns 1–3) and FE (columns 2–4) estimates of specification (1), with, respectively, the number of chronic diseases (columns 1–2) and a dummy if affected by at least one diseases (columns 3–4) as dependent variables. Region FE include regions in Austria, Belgium, Czech Republic, Denmark, Estonia, France, Germany, Greece, Italy, The Netherlands, Poland, Slovenia, Spain, and Sweden. Wave FE include waves 1, 2, 4, 5, and 6 covering the period 2004–2015. Robust standard errors clustered at regional level in parenthesis

***p < 0:01; **p < 0:05; *p < 0:1

### Heterogeneity analysis

In what follows, we assess whether corruption has a heterogeneous effect on health outcomes depending on (1) factors related to the respondent’s country of residence, (2) individual socio-demographic characteristics, and (3) type of morbidity.

### Country GDP and health expenditure per capita

The effects of corruption on healthcare outcomes may be particularly detrimental for individuals living in low-income countries, for a number of interconnected reasons. First, low-GDP per capita is generally associated with low public healthcare expenditure, which has been shown to be correlated with poor health outcomes (Becchetti et al. 2017). At the same time, professionals operating in low-expenditure healthcare systems generally earn relatively low salaries, especially when the healthcare system is financed through general taxation (European Commission 2013). In healthcare systems characterised by compulsory insurance schemes, however, the most relevant forms of corruption are billing frauds, excessive medical treatments, and diversion of funds. Thus, the scarcity of resources reduces access to healthcare especially for individuals who are less wealthy, and increases the incentive for healthcare professionals to ask for informal payments, i.e. bribes, in exchange for access to healthcare. Thus, the costs of corruption in terms of chronic diseases would be larger in countries where individuals are relatively poorer and healthcare expenditure lower.

To test this hypothesis empirically, we create two dummies, High_GDP_*ci*_,_*t*_ and High_EXP_*ci*_,_*t*_, which equal 1 if respondent *i*’s country of residence in wave *t* is above the median GDP per capita and the median public healthcare expenditure per capita, respectively. We use data on GDP from Eurostat and public healthcare expenditures from OECD. We then split our dataset accordingly (see ESM-B for the groups of countries) and run the full specification of (1) separately on each sub-sample.

The effect of CPI on both variables is always negative but significant only for respondents living in countries with below-median GDP and public healthcare expenditure per capita (Table 4). Interestingly, the absolute value of the estimated coefficients for low-GDP and low-expenditure countries is almost twice as large as the one estimated for the whole sample, suggesting that these variables are crucial in determining the effects of corruption on health outcomes. These findings support our claim that the effect of corruption on morbidity is heterogeneous and depends on a country’s wealth and healthcare policies.

**Table 4:** The heterogeneous effect of Corruption Perception Index

Dependent variable	(1)	(2)	Mean	Obs.
Number of chronic diseases	OLS	FE		
*Country characteristics*				
Low p.c. GDP	− 0.127***	− 0.071**	1.34	50,209
	(0.0282)	(0.0267)		
High p.c. GDP	− 0.0184	− 0.0314	1.108	111,432
	(0.0237)	(0.0207)		
Low expenditure	− 0.128***	− 0.0986***	1.323	56,995
	(0.0292)	(0.026)		
High expenditure	− 0.0136	− 0.0259	1.1	104,646
	(0.0242)	(0.0214)		
*Individual characteristics*				
Low HH income	− 0.0853***	− 0.098***	1.32	80,883
	(0.0274)	(0.0281)		
High HH income	− 0.0442	− 0.0425*	1.038	80,758
	(0.0279)	(0.025)		
Aged 50–65	− 0.0381*	− 0.0366	0.864	81,173
	(0.0226)	(0.0226)		
Aged 66+	− 0.0958***	− 0.0923***	1.497	80,468
	(0.0334)	(0.0339)		
Male	− 0.0588*	− 0.058**	1.191	72,182
	(0.0305)	(0.0287)		
Female	− 0.0777***	− 0.078***	1.169	89,459
	(0.0246)	(0.0241)		
Low education	− 0.0789***	− 0.0562**	1.315	89,638
	(0.03)	(0.0264)		
High education	− 0.0836***	− 0.0463	1.009	72,003
	(0.026)	(0.0293)		

Europe, data from 2004 to 2015. p.c. *GDP* per capita Gross Domestic Product, *OLS* ordinary least squares, *FE* fixed effects, *CPI* Corruption Perception Index. Pooled OLS (column 1) and FE (column 2) estimates of specification (1), using the number of diseases as outcome variable. The sample is split, respectively, by p.c. GDP, p.c. public healthcare expenditure, household income, age, gender, and highest educational attainment

Robust standard errors clustered at the regional level in parenthesis

***p < 0.01; **p < 0.05; *p < 0.1

### The role of individual characteristics

We exploit the detailed information provided by SHARE and investigate additional channels through which corruption may heterogeneously affect health. In particular, we look at how people who differ in gender, age, education, and income are affected by corruption. These identify key variables for corruption attitudes as well as standard characteristics for socio-economic status. Therefore, we split the sample depending whether respondents were above or below the median value of each characteristic considered. Table 4 reports findings from the full specification of (1) on each sub-sample.

The effect of CPI remains negative in both sub-samples but significant only for individuals whose household income is lower than the country’s median in that specific wave (Table 4). This result is particularly interesting if we consider that relatively low-income people are likely to translate to a reduced healthcare access, especially in countries where healthcare is not publicly financed. As for other socio-demographic dimensions, we find that females, the elders, and low educated people exhibit a stronger and more stable negative pattern with corruption when compared to their counterparts.

### Corruption and categories of chronic disease

We also investigate the effect of CPI on the respondent’s probability of being affected by a specific category of chronic disease. This analysis may help us understanding whether corruption has a heterogeneous effect depending on the morbidity. While we do not have any ex-ante hypotheses on the link between corruption and type of disease, we want to investigate whether it may be explained by a kind of severity of illness. To investigate this, we estimate (1) using as a dependent variable a dummy equal to 1 if the respondent is affected in wave *t* by at least one of the diseases included in the categories defined in ESM-B, i.e. cancer, cardiovascular, age, respiratory, ulcer, and hip or femur fracture.

The effect of CPI is negative for all categories, in line with our aggregate estimates, and strongly significant for both cardiovascular diseases and ulcer (Table 5). The absence of a significant effect on the other diseases may be justified by their rarity in our sample.

**Table 5:** The impact of Corruption Perception Index on the probability of suffering from a category of diseases

	(1) OLS	(2) FE	Mean	Obs.
Age-related	− 0.0036	− 0.0062	0.089	161,641
	(0.0047)	(0.0045)		
Cancer	− 0.001	− 0.0017	0.392	161,641
	(0.0025)	(0.0029)		
Cardiovascular	− 0.018***	− 0.0162***	0.564	161,641
	(0.0059)	(0.0057)		
Hip fracture	− 0.0024	− 0.002	0.018	161,641
	(0.0015)	(0.0016)		
Lung disease	− 0.0023	− 0.005	0.063	161,641
	(0.0036)	(0.0032)		
Ulcer	− 0.0106**	− 0.012***	0.047	161,641
	(0.053)	(0.0044)		

Europe, data from 2004 to 2015. *OLS* ordinary least squares, *FE* fixed effects, *CPI* Corruption Perception Index. Pooled OLS (column 1) and FE (column 2) estimates of specification (1), using the occurrence of a specific category of disease as outcome variable

All results are confirmed when we use CoC as a measure of corruption (see ESM-A).

### Perception of corruption in different sectors

To further explore the corruption–health link, we implement an analysis with sectorial corruption perception. Eurobarometer data measure respondents’ beliefs about corruption in the following sectors: public healthcare, police, judicial courts, national politicians, public tenders, and the concession of building and business permits. ESM-A shows results from estimates as in (1) where CPI is replaced by national average of corruption perception in each sector. Corruption in healthcare is strongly and positively associated with the number of chronic diseases. This strengthens our main results suggesting healthcare as a possible channel of the detrimental effect of corruption on individual health. We find a similar effect in the politics sector, even though smaller than the one in the healthcare. Interestingly, among the other sectors coefficients remain positive but not significant. This is consistent with the hypothesis that only corruption that may interact with healthcare sector is detrimental for individual health.

## Discussion

The literature on corruption and health has so far focussed mainly on the effects of corruption in developing countries on aggregate health outcomes like mortality indicators (Gupta et al. 2000; Muldoon et al. 2011; Nadpara and Samanta 2015). Surprisingly, we did not find any contribution assessing these links for high-income countries and with individual morbidity indicators.

With an analysis on Europeans aged 50+, our findings show that national corruption is positively associated with the number of chronic diseases and the probability of being affected by at least one disease. Estimates are robust to the inclusion of a wide range of socio-demographic and healthy lifestyle individual factors, as well as the use of alternative proxies for corruption.

In line with the literature on developing countries (Gupta et al. 2000; Nadpara and Samanta 2015), the effect is mainly concentrated among poorer countries. In other words, poor European countries and developing countries do exhibit a similar pattern, while rich European countries do not. Also, the effect is more prevalent for cardiovascular diseases and ulcers. This makes our study particularly relevant if we consider that cardiovascular diseases represent one of the main causes of death worldwide (32% in 2015, Wang et al. 2016).

Our study is extremely informative for policy implications in at least three directions. First, we provide a clear evidence of the detrimental effect of corruption on health outcomes, therefore rejecting the hypothesis that corruption serves as an informal stimulus to increase the quantity of healthcare services (Leff 1964; Huntington 2006; Méon and Weill 2010) and reinforcing the fight against corruption as a possible tool to improve public health (Mackey et al. 2016). As defined by Acheson (1998), public health is “the art and science of preventing disease, prolonging life, and promoting health through the organised efforts of society”. Therefore, our study advances the under-researched debate on the link between social behaviour and population health (Judge et al. 2011). Second, we show that the health of specific sub-groups such as females and those with poor socio-economic status is more damaged by corruption than that of their counterparts. Possible explanations may arise from gender differences in corruption attitudes (Swamy et al. 2001; Dollar et al. 2001) as well as the fact that individuals with poor socio-economic status are less likely to be involved in corruptive practices because of limited resources. As a result, decreasing corruption would be possibly entailing not only a decrease in the population health as a whole but also a reduction in health inequality. Third, our findings highlight the relation between highly corrupted countries and cardiovascular diseases, the leading cause of death in our countries (Wang et al. 2016). It would be interesting for further research to understand whether disease-specific relations are explained by differences in healthcare systems or through the link with other non-observable factors [for instance, other clinical factors such as antimicrobial resistance, Collignon et al. (2018)].

The relation suggests that corruption is detrimental for health, and this may be explained with a reduction in the quantity and the quality of healthcare services. However, our analysis does not explicitly test this channel. We acknowledge this limitation and we suggest future research to disentangle the role of healthcare in the corruption–health nexus, for instance with data on healthcare personnel wages and services costs. Our analysis does not address some caveats related with corruption in healthcare either, such as the type of corruption and the causal nexus between corruption and health outcomes. As for the type of corruption, our measure includes all corruption activities, ranging from grand corruption to petty corruption in the public sector. With more detailed data, a further analysis assessing heterogeneity across different types of corruption would be worth. In particular, future research could help in explaining whether the link is between overall corruption and health (for instance, though, individual distress) or sectorial corruption (and, if so, through less healthcare quantity and quality and/or differences in public and private healthcare). Regarding the causality nexus, we might think both as corruption deteriorating healthcare quality and ultimately reducing individual health outcomes, as well as sick people demanding for more or better healthcare services through corruption practices. With our results, we do not claim any causality direction, even though our corruption measures capture also corruption unrelated with healthcare sector, which is unlikely to be caused by any diseases. In addition, we find the link between corruption and health stronger for relatively poorer individuals, who have more constraints on corruption practices like bribes (Davis et al. 2004; Corrado et al. 2017). We are also aware of the limitations embedded in most of the corruption indices, though we strongly believe that our empirical evidence provides the literature with very important insights on a relevant topic for high-income countries.

## Electronic supplementary material

Below is the link to the electronic supplementary material.

Supplementary material 1 (DOCX 193 kb)
