# Supplementary material for: The relationship between corruption and chronic diseases: evidence from Europeans aged 50 years and older
Source: Int J Public Health. 2020 Mar 26;65(3):345–55. doi: 10.1007/s00038-020-01347-w (PMC7183492; doi:10.1007/s00038-020-01347-w)
Supplement: Supplementary file 1 — Supplementary material 1 (DOCX 193 kb) [file 38_2020_1347_MOESM1_ESM.docx]

**Electronic Supplementary Material (ESA)**

Journal name: International Journal of Public Health

Title: The relationship between corruption and chronic diseases: Evidence from Europeans aged 50 years and older

# Appendix A (ESA-A). Additional tables and figures

Table A1. Descriptive Statistics.

| **Variable** | **Mean** | **Std. Dev.** | **Min** | **p25** | **p50** | **p75** | **Max** |
| --- | --- | --- | --- | --- | --- | --- | --- |
| *Corruption, country, and wave characteristics* | | | | | | | |
| *CPI* | 6.959 | 1.57 | 3.7 | 5.8 | 7.1 | 8.1 | 9.5 |
| *CoC* | 1.3 | 0.713 | 0 | 0.6 | 1.4 | 1.8 | 2.5 |
| *Country* |  |  |  |  |  |  |  |
| Austria | 0.072 | 0.258 | 0 | 0 | 0 | 0 | 1 |
| Belgium | 0.115 | 0.319 | 0 | 0 | 0 | 0 | 1 |
| Czech Republic | 0.096 | 0.294 | 0 | 0 | 0 | 0 | 1 |
| Denmark | 0.075 | 0.264 | 0 | 0 | 0 | 0 | 1 |
| Estonia | 0.092 | 0.29 | 0 | 0 | 0 | 0 | 1 |
| Germany | 0.086 | 0.281 | 0 | 0 | 0 | 0 | 1 |
| Greece | 0.027 | 0.161 | 0 | 0 | 0 | 0 | 1 |
| France | 0.096 | 0.295 | 0 | 0 | 0 | 0 | 1 |
| Italy | 0.086 | 0.281 | 0 | 0 | 0 | 0 | 1 |
| Netherlands | 0.06 | 0.238 | 0 | 0 | 0 | 0 | 1 |
| Poland | 0.023 | 0.15 | 0 | 0 | 0 | 0 | 1 |
| Slovenia | 0.042 | 0.2 | 0 | 0 | 0 | 0 | 1 |
| Spain | 0.055 | 0.228 | 0 | 0 | 0 | 0 | 1 |
| Sweden | 0.074 | 0.262 | 0 | 0 | 0 | 0 | 1 |
| *Wave* |  |  |  |  |  |  |  |
| 1 (2004-2005) | 0.101 | 0.302 | 0 | 0 | 0 | 0 | 1 |
| 2 (2006-2007) | 0.164 | 0.37 | 0 | 0 | 0 | 0 | 1 |
| 4 (2011-2012) | 0.249 | 0.433 | 0 | 0 | 0 | 1 | 1 |
| 5 (2013) | 0.283 | 0.45 | 0 | 0 | 0 | 1 | 1 |
| 6 (2015) | 0.202 | 0.402 | 0 | 0 | 0 | 0 | 1 |
| *Individual characteristics* | | | | | | | |
| Age | 66.53 | 9.804 | 50 | 58.6 | 65.5 | 73.5 | 104.5 |
| Female | 0.553 | 0.497 | 0 | 0 | 1 | 1 | 1 |
| Years of education | 11.017 | 4.166 | 0 | 8 | 11 | 14 | 25 |
| Log Income | 9.802 | 1.305 | 0 | 9.394 | 9.928 | 10.458 | 15.997 |
| Children | 2.161 | 1.322 | 0 | 1 | 2 | 3 | 10 |
| *Job* |  |  |  |  |  |  |  |
| Retired | 0.582 | 0.493 | 0 | 0 | 1 | 1 | 1 |
| Employed | 0.258 | 0.438 | 0 | 0 | 0 | 1 | 1 |
| Unemployed | 0.028 | 0.165 | 0 | 0 | 0 | 0 | 1 |
| Sick | 0.034 | 0.181 | 0 | 0 | 0 | 0 | 1 |
| Homemaker | 0.086 | 0.28 | 0 | 0 | 0 | 0 | 1 |
| Other | 0.011 | 0.107 | 0 | 0 | 0 | 0 | 1 |
| *Marital Status* |  |  |  |  |  |  |  |
| Married | 0.71 | 0.454 | 0 | 0 | 1 | 1 | 1 |
| Not married | 0.054 | 0.227 | 0 | 0 | 0 | 0 | 1 |
| Divorced | 0.085 | 0.28 | 0 | 0 | 0 | 0 | 1 |
| Widowed | 0.15 | 0.357 | 0 | 0 | 0 | 0 | 1 |
| *Living area* |  |  |  |  |  |  |  |
| City | 0.434 | 0.496 | 0 | 0 | 0 | 0 | 1 |
| Small city | 0.252 | 0.434 | 0 | 0 | 0 | 1 | 1 |
| Rural | 0.314 | 0.464 | 0 | 0 | 0 | 1 | 1 |
| *Healthy Lifestyle* |  |  |  |  |  |  |  |
| Ever Smoked Daily | 0.474 | 0.499 | 0 | 0 | 0 | 1 | 1 |
| *Alcohol Consumption* |  |  |  |  |  |  |  |
| Never | 0.294 | 0.456 | 0 | 0 | 0 | 1 | 1 |
| Sometimes | 0.229 | 0.42 | 0 | 0 | 0 | 0 | 1 |
| Often | 0.478 | 0.499 | 0 | 0 | 0 | 1 | 1 |
| *BMI Class* |  |  |  |  |  |  |  |
| Underweight | 0.012 | 0.108 | 0 | 0 | 0 | 0 | 1 |
| Normal weight | 0.361 | 0.48 | 0 | 0 | 0 | 1 | 1 |
| Overweight | 0.416 | 0.493 | 0 | 0 | 0 | 1 | 1 |
| Obese | 0.212 | 0.408 | 0 | 0 | 0 | 0 | 1 |
| *Sport* |  |  |  |  |  |  |  |
| Never | 0.43 | 0.495 | 0 | 0 | 0 | 1 | 1 |
| Sometimes | 0.229 | 0.42 | 0 | 0 | 0 | 0 | 1 |
| Often | 0.342 | 0.474 | 0 | 0 | 0 | 1 | 1 |
| *Health outcomes* | | | | | | | |
| No. Diseases | 1.179 | 1.224 | 0 | 0 | 1 | 2 | 10 |
| At least one Disease | 0.638 | 0.48 | 0 | 0 | 1 | 1 | 1 |
| Age-related | 0.089 | 0.285 | 0 | 0 | 0 | 0 | 1 |
| Cataracts | 0.083 | 0.276 | 0 | 0 | 0 | 0 | 1 |
| Parkinson | 0.007 | 0.086 | 0 | 0 | 0 | 0 | 1 |
| Cancer | 0.051 | 0.221 | 0 | 0 | 0 | 0 | 1 |
| Cardiovascular | 0.564 | 0.496 | 0 | 0 | 1 | 1 | 1 |
| Blood Pressure | 0.392 | 0.488 | 0 | 0 | 0 | 0 | 1 |
| Cholesterol | 0.229 | 0.42 | 0 | 0 | 0 | 0 | 1 |
| Diabetes | 0.121 | 0.326 | 0 | 0 | 0 | 0 | 1 |
| Hearth Attack | 0.126 | 0.332 | 0 | 0 | 0 | 0 | 1 |
| Stroke | 0.039 | 0.038 | 0 | 0 | 0 | 0 | 1 |
| Hip Fracture | 0.018 | 0.135 | 0 | 0 | 0 | 0 | 1 |
| Lung Disease | 0.063 | 0.243 | 0 | 0 | 0 | 0 | 1 |
| Ulcer | 0.047 | 0.212 | 0 | 0 | 0 | 0 | 1 |
| Notes: Europe, data from 2004 to 2015. CPI = Corruption Perception Index; CoC = Control of Corruption Index; BMI = Body Mass Index. p25, p50, p75 show respectively the 25^th^, 50^th^, and 75^th^ percentile. Min and Max show respectively the minimum and the maximum values. | | | | | | | |

Figure 1. Chronic diseases and Corruption Perception Index during 2004-2015, by country.

(a)

(b)


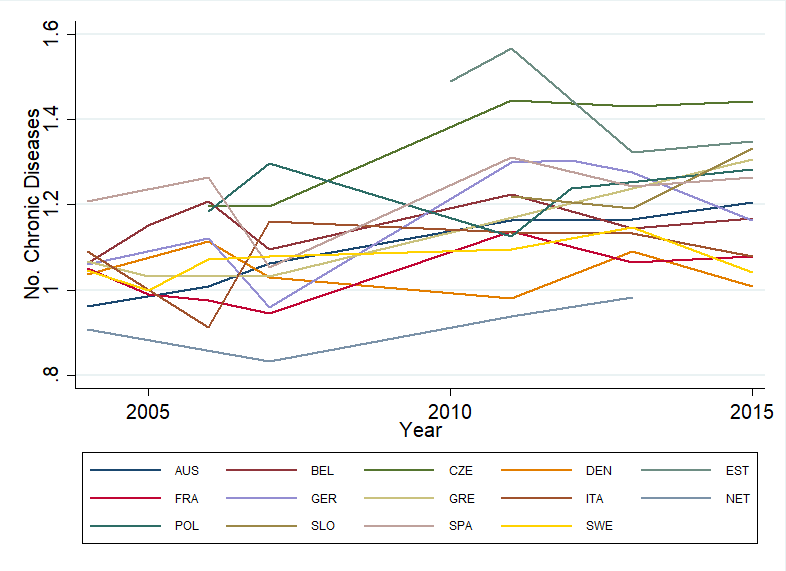

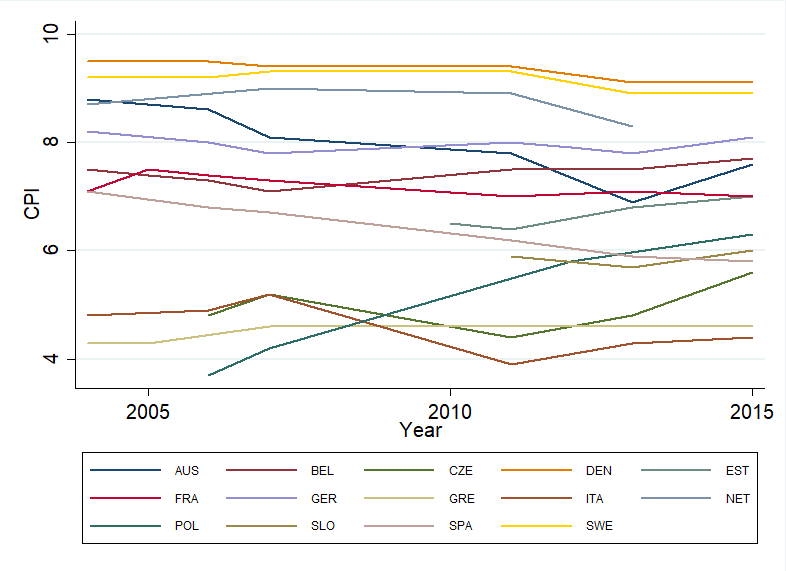


Notes: AUS = Austria, BEL = Belgium, CZE = Czech Republic, DEN = Denmark, EST = Estonia, FRA = France, GER = Germany, GRE = Greece, ITA = Italy, NET = The Netherlands, POL = Poland, SLO – Slovenia, SPA = Spain, SWE = Sweden. Year covers the period 2004-2015.

Figure A2: Number and probability of chronic diseases for Control of Corruption quartiles.

(a)

(b)


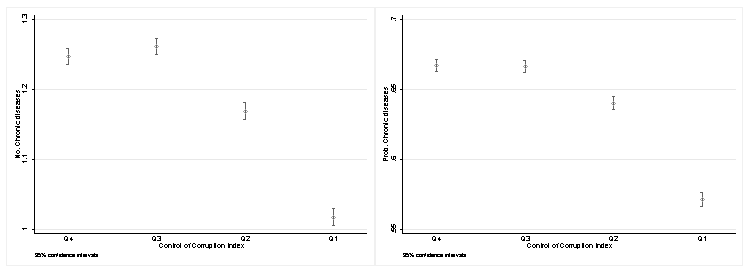


Notes. Europe, data from 2004 to 2015. Average number of chronic diseases (a) and the probability of being affected by at least one chronic diseases (b) with 95% confidence intervals, by Control of Corruption index quartiles. Q1, Q2, Q3, and Q4 represent Control of Corruption quartiles, from the the highest (the least corrupted countries) to the lowest (the most corrupted countries).

Table A2. The impact of Corruption Perception Index on the number of chronic diseases (complete).

| *Dependent Variable:* | (1) | (2) |
| --- | --- | --- |
| *No. of chronic diseases* | OLS | FE |
| CPI | -0.0696*** | -0.0691*** |
|  | (0.026) | (0.0249) |
| Age | 0.0261*** | 0.0247 |
|  | (0.0008) | (0.0259) |
| Female | -0.0514*** | - |
|  | (0.0145) |  |
| Log Income | 0.0049 | 0.0077** |
|  | (0.0034) | (0.0034) |
| Education Years | -0.0113*** | - |
|  | (0.0016) |  |
| No. of Children | 0.0007 | 0.0226** |
|  | (0.0046) | (0.0093) |
| *Job (Ref=Retired):* |  |  |
| Employed | -0.234*** | -0.0492*** |
|  | (0.0129) | (0.0116) |
| Unemployed | -0.171*** | -0.0528*** |
|  | (0.0259) | (0.0153) |
| Sick | 0.374*** | 0.1*** |
|  | (0.0226) | (0.0265) |
| Homemaker | -0.084*** | -0.0781*** |
|  | (0.0206) | (0.0192) |
| Other | -0.039 | -0.025 |
|  | (0.0293) | (0.0232) |
| *Marital Status (Ref=Married)*: |  |  |
| Not Married | -0.0432 | -0.0475 |
|  | (0.0283) | (0.104) |
| Divorced | 0.002 | -0.0005 |
|  | (0.0182) | (0.0431) |
| Widowed | 0.0215 | 0.0144 |
|  | (0.0293) | (0.0229) |
| *Living Area (Ref=City)*: |  |  |
| Small City | -0.0267*** | -0.0028 |
|  | (0.0094) | (0.0133) |
| Rural | -0.0369** | 0.0044 |
|  | (0.0159) | (0.0176) |
| Ever Smoked Daily | 0.123*** | 0.014 |
|  | (0.0096) | (0.171) |
| *Alcohol Consumption (Ref=Never)*: |  |  |
| Sometimes | -0.102*** | 0.0206** |
|  | (0.0196) | (0.0103) |
| Often | -0.158*** | 0.0095 |
|  | (0.0187) | (0.0134) |
| *BMI Class (Ref=Underweight)*: |  |  |
| Normal Weight | -0.0348 | -0.0434 |
|  | (0.0315) | (0.0342) |
| Overweight | 0.229*** | 0.0122 |
|  | (0.0075) | (0.0101) |
| Obese | 0.58*** | 0.112*** |
|  | (0.0171) | (0.0226) |
| *Sport (Ref=Never)*: |  |  |
| Sometimes | -0.245**** | -0.0591*** |
|  | (0.0182) | (0.0108) |
| Often | -0.307*** | -0.0589*** |
|  | (0.0219) | (0.0101) |
| Region FE | Yes | No |
| Wave FE | Yes | Yes |
| Mean | 1.1789 | 1.1789 |
| Observations | 161,641 | 161,641 |
| R-squared | 0.175 | 0.12 |
| Notes. Europe, data from 2004 to 2015. OLS = Ordinary Least Squares; FE = Fixed Effects; CPI = Corruption Perception Index; BMI = Body Mass Index.  Pooled OLS and FE estimates of specification (1).  Robust standard errors clustered at regional level in parenthesis. *** p < 0.01, ** p < 0.05, * p < 0.1. | | |

Table A3. The impact of Corruption Perception Index on the probability of suffering from at least one chronic disease.

| *Dependent Variable:* | (1) | (2) | (3) | (4) | (5) | (6) | (7) | (8) |
| --- | --- | --- | --- | --- | --- | --- | --- | --- |
| At least one chronic diseases | Logit | Logit | Logit | Logit | Probit | Probit | Probit | Probit |
| CPI | -067** | -0.0696** | -0.0901** | -0.0911** | -0.0421** | -0.0439** | -0.0556*** | -0.0563*** |
|  | (0.0339) | (0.0343) | (0.0356) | (0.0358) | (0.0203) | (0.0206) | (0.021) | (0.0212) |
|  |  |  |  |  |  |  |  |  |
| Age | 0.062*** | 0.0456*** | 0.0594*** | 0.047*** | 0.0369*** | 0.0268*** | 0.0351*** | 0.0275*** |
|  | (0.0015) | (0.0014) | (0.0016) | (0.0014) | (0.0009) | (0.0008) | (0.0009) | (0.0008) |
| Female | -0.0706*** | -0.110*** | -0.0574*** | -0.0751*** | -0.041*** | -0.0643*** | -0.0341*** | -0.0446*** |
|  | (0.0238) | (0.03) | (0.0215) | (0.0257) | (0.0147) | (0.0186) | (0.0131) | (0.0156) |
|  |  |  |  |  |  |  |  |  |
| Region FE | Yes | Yes | Yes | Yes | Yes | Yes | Yes | Yes |
| Wave FE | Yes | Yes | Yes | Yes | Yes | Yes | Yes | Yes |
| Socio-Demographics | No | Yes | No | Yes | No | Yes | No | Yes |
| Healthy Lifestyle | No | No | Yes | Yes | No | No | Yes | Yes |
|  |  |  |  |  |  |  |  |  |
| Mean | 0.6383 | 0.6383 | 0.6383 | 0.6383 | 0.6383 | 0.6383 | 0.6383 | 0.6383 |
| Observations | 161,641 | 161,641 | 161,641 | 161,641 | 161,641 | 161,641 | 161,641 | 161,641 |
| Pseudo R-squared | 0.067 | 0.077 | 0.099 | 0.105 | 0.066 | 0.077 | 0.099 | 0.104 |
| Notes. Europe, data from 2004 to 2015. CPI = Corruption Perception Index; FE = Fixed Effects. Logit (columns 1-4) and Probit (columns 5-8) estimates of specification (1), with a dummy if affected by at least one diseases as dependent variable.  Robust standard errors clustered at regional level in parenthesis. *** p < 0:01, ** p < 0:05, * p < 0:1. | | | | | | | | |

Table A4. The impact of Corruption Perception Index on the probability of suffering from two, three, and four chronic diseases.

|  | OLS | FE | Mean | Obs. |
| --- | --- | --- | --- | --- |
| *Dependent Variable:* |  |  |  |  |
| Suffering from at least |  |  |  |  |
| Two | -0.0193*** | -0.0421** | 0.327 | 161,641 |
|  | (0.0065) | (0.0203) |  |  |
| Three | -0.0192** | -0.0176** | 0.141 | 161,641 |
|  | (0.0077) | (0.008) |  |  |
| Four | -0.0102** | -0.01** | 0.051 | 161,641 |
|  | (0.0042) | (0.004) |  |  |
| Notes. Europe, data from 2004 to 2015. OLS = Ordinary Least Squares; FE = Fixed Effects; Obs. = Observations. Pooled OLS (column 1) and fixed-effects (column 2) estimates of specification (1), with each line showing health coefficient for the probability of suffering an additional disease when suffering already respectively 1, 2, and 3.  Robust standard errors clustered at the regional level in parenthesis. *** p < 0.01, ** p < 0.05, * p < 0.1. | | | | |

Table A5. The impact of Corruption Perception Index on the number and probability of chronic diseases, sample split by per capita Gross Domestic Product.

| *Dependent Variable:* | No. of chronic diseases | | | | At least one chronic disease | | | |
| --- | --- | --- | --- | --- | --- | --- | --- | --- |
|  | Low p.c. GDP | | High p.c. GDP | | Low p.c. GDP | | High p.c. GDP | |
|  | (1) | (2) | (3) | (4) | (5) | (6) | (7) | (8) |
|  | OLS | FE | OLS | FE | OLS | FE | OLS | FE |
| CPI | -0.127*** | -0.071** | -0.0184 | -0.0314 | -0.0313*** | -0.0203** | -0.0083 | -0.0127* |
|  | (0.0282) | (0.0267) | (0.0237) | (0.0207) | (0.0089) | (0.0078) | (0.0082) | (0.0076) |
|  |  |  |  |  |  |  |  |  |
| Age | 0.0255*** | -0.054** | 0.0265*** | 0.0766*** | 0.0087*** | -0.016** | 0.0093*** | 0.0258** |
|  | (0.0012) | (0.0265) | (0.001) | (0.0266) | (0.0004) | (0.0079) | (0.0003) | (0.0109) |
|  |  |  |  |  |  |  |  |  |
| Female | -0.0362* | - | -0.061*** | - | -0.007 | - | -0.0213*** | - |
|  | (0.0209) |  | (0.0171) |  | (0.0056) |  | (0.0067) |  |
|  |  |  |  |  |  |  |  |  |
| Region FE | Yes | No | Yes | No | Yes | No | Yes | No |
| Wave FE | Yes | Yes | Yes | Yes | Yes | Yes | Yes | Yes |
| Socio-Demographics | Yes | Yes | Yes | Yes | Yes | Yes | Yes | Yes |
| Healthy Lifestyle | Yes | Yes | Yes | Yes | Yes | Yes | Yes | Yes |
|  |  |  |  |  |  |  |  |  |
| Mean | 1.342 | 1.34 | 1.108 | 1.108 | 0.6913 | 0.6913 | 0.6151 | 0.6151 |
| Observations | 50,209 | 50,209 | 111,432 | 111,432 | 50,209 | 50,209 | 111,432 | 111,432 |
| R-squared | 0.177 | 0.037 | 0.167 | 0.092 | 0.131 | 0.027 | 0.124 | 0.076 |
| Notes. Europe, data from 2004 to 2015. p.c. GDP = per capita Gross Domestic Product; OLS = Ordinary Least Squares; FE = Fixed Effects; CPI = Corruption Perception Index. Pooled OLS (columns 1-3-5-7) and FE (columns 2-4-6-8) estimates of specification (1), using the number and the probability of diseases as outcome variables. The sample is split by p.c. GDP.  Robust standard errors clustered at the regional level in parenthesis. *** p < 0.01, ** p < 0.05, * p < 0.1. | | | | | | | | |

Table A6. The impact of Corruption Perception Index on the number and probability of chronic diseases, sample split by healthcare expenditure.

| *Dependent Variable:* | No. of chronic diseases | | | | At least one chronic disease | | | |
| --- | --- | --- | --- | --- | --- | --- | --- | --- |
|  | Low Expenditure | | High Expenditure | | Low Expenditure | | High Expenditure | |
|  | (1) | (2) | (3) | (4) | (5) | (6) | (7) | (8) |
|  | OLS | FE | OLS | FE | OLS | FE | OLS | FE |
| CPI | -0.128*** | -0.0986*** | -0.0136 | -0.0259 | -0.0324*** | -0.0261*** | -0.0065 | -0.0113 |
|  | (0.0292) | (0.0242) | (0.0260) | (0.0214) | (0.0082) | (0.0065) | (0.0083) | (0.0078) |
|  |  |  |  |  |  |  |  |  |
| Age | 0.025*** | -0.0591** | 0.0269*** | 0.084*** | 0.0086*** | -0.0162* | 0.0094*** | 0.0261** |
|  | (0.0012) | (0.029) | (0.001) | (0.0271) | (0.0004) | (0.0091) | (0.0003) | (0.0112) |
|  |  |  |  |  |  |  |  |  |
| Female | -0.0406** | - | -0.0593*** | - | -0.0081 | - | -0.0212*** | - |
|  | (0.0202) |  | (0.0178) |  | (0.0056) |  | (0.0069) |  |
|  |  |  |  |  |  |  |  |  |
| Region FE | Yes | No | Yes | No | Yes | No | Yes | No |
| Wave FE | Yes | Yes | Yes | Yes | Yes | Yes | Yes | Yes |
| Socio-Demographics | Yes | Yes | Yes | Yes | Yes | Yes | Yes | Yes |
| Healthy Lifestyle | Yes | Yes | Yes | Yes | Yes | Yes | Yes | Yes |
|  |  |  |  |  |  |  |  |  |
| Mean | 1.323 | 1.323 | 1.1 | 1.1 | 0.6875 | 0.6875 | 0.6115 | 0.6115 |
| Observations | 56,995 | 56,995 | 104,646 | 104,646 | 56,995 | 56,995 | 104,646 | 104,646 |
| R-squared | 0.178 | 0.042 | 0.165 | 0.089 | 0.131 | 0.027 | 0.124 | 0.076 |
| Notes. Europe, data from 2004 to 2015. OLS = Ordinary Least Squares; FE = Fixed Effects; CPI = Corruption Perception Index. Pooled OLS (columns 1-3-5-7) and FE (columns 2-4-6-8) estimates of specification (1), using the number and the probability of diseases as outcome variables. The sample is split by healthcare expenditure.  Robust standard errors clustered at the regional level in parenthesis. *** p < 0.01, ** p < 0.05, * p < 0.1. | | | | | | | | |

Table A7. Heterogeneous effect of Control of Corruption. Country characteristics.

| *Dependent Variable:* |  |  |  |  |
| --- | --- | --- | --- | --- |
| Number of chronic diseases | OLS | FE | Mean | Obs. |
| Low p.c. GDP | -0.728*** | -0.773*** | 1.34 | 50,209 |
|  | (0.0977) | (0.094) |  |  |
| High p.c. GDP | -0.0826 | -0.137* | 1.108 | 111,432 |
|  | (0.0756) | (0.0729) |  |  |
| Low Expenditure | -0.424*** | -0.401*** | 1.323 | 56,995 |
|  | (0.0795) | (0.08) |  |  |
| High Expenditure | -0.062 | -0.0928 | 1.1 | 104,646 |
|  | (0.0714) | (0.0787) |  |  |
| Notes. Europe, data from 2004 to 2015. OLS = Ordinary Least Squares; FE = Fixed Effects; Obs. = Observations; p.c. GDP = per capita Gross Domestic Product. Pooled OLS (column 1) and FE (column 2) estimates of specification (1). Socio-demographics include years of education, household income, job status, marital status, place of living, and the number of children. Healthy lifestyle include smoking behaviour, alcohol consumption, sport engagement, and the Body Mass Index category. The sample  is split by median p.c. GDP and government healthcare expenditure at country level. Countries in the low p.c. GDP group are Czech Republic, Estonia, Greece, Poland, Slovenia, and Spain (for the years 2004-2012). Countries in the low per capita healthcare expenditure group are Czech Republic, Estonia, Greece, Italy (in 2015), Poland, Slovenia, and Spain.  Robust standard errors clustered at the regional level in parenthesis. *** p < 0.01, ** p < 0.05, * p < 0.1. | | | | |

Table A8. The impact of Corruption Perception Index on the number and probability of chronic diseases, sample split by household income.

| *Dependent Variable:* | No. of chronic diseases | | | | At least one chronic disease | | | |
| --- | --- | --- | --- | --- | --- | --- | --- | --- |
|  | Low HH income | | High HH income | | Low HH income | | High HH income | |
|  | (1) | (2) | (3) | (4) | (5) | (6) | (7) | (8) |
|  | OLS | FE | OLS | FE | OLS | FE | OLS | FE |
| CPI | -0.0853*** | -0.098*** | -0.0442 | -0.0425* | -0.0224*** | -0.0302*** | -0.0103 | -0.0087 |
|  | (0.0274) | (0.0279) | (0.0281) | (0.025) | (0.0078) | (0.0079) | (0.0079) | (0.0078) |
|  |  |  |  |  |  |  |  |  |
| Age | 0.0237*** | 0.0296 | 0.0285*** | 0.0389 | 0.0076*** | 0.0163 | 0.0107*** | 0.0094 |
|  | (0.0009) | (0.0331) | (0.001) | (0.0291) | (0.0003) | (0.01) | (0.0003) | (0.011) |
|  |  |  |  |  |  |  |  |  |
| Female | -0.025 | - | -0.0744*** | - | -0.0067 | - | -0.0233*** | - |
|  | (0.0201) |  | (0.0126) |  | (0.0064) |  | (0.0056) |  |
|  |  |  |  |  |  |  |  |  |
| Region FE | Yes | No | Yes | No | Yes | No | Yes | No |
| Wave FE | Yes | Yes | Yes | Yes | Yes | Yes | Yes | Yes |
| Socio-Demographics | Yes | Yes | Yes | Yes | Yes | Yes | Yes | Yes |
| Healthy Lifestyle | Yes | Yes | Yes | Yes | Yes | Yes | Yes | Yes |
|  |  |  |  |  |  |  |  |  |
| Mean | 1.32 | 1.32 | 1.038 | 1.038 | 0.681 | 0.681 | 0.596 | 0.596 |
| Observations | 80,883 | 80,883 | 80,758 | 80,758 | 80,883 | 80,883 | 80,758 | 80,758 |
| R-squared | 0.154 | 0.052 | 0.183 | 0.124 | 0.117 | 0.064 | 0.135 | 0.086 |
| Notes. Europe, data from 2004 to 2015. HH = Household; OLS = Ordinary Least Squares; FE = Fixed Effects; CPI = Corruption Perception Index. Pooled OLS (columns 1-3-5-7) and FE (columns 2-4-6-8) estimates of specification (1), using the number and the probability of diseases as outcome variables. The sample is split by household income.  Robust standard errors clustered at the regional level in parenthesis. *** p < 0.01, ** p < 0.05, * p < 0.1. | | | | | | | | |

Table A9. The impact of Corruption Perception Index on the number and probability of chronic diseases, sample split by age.

| *Dependent Variable:* | No. of chronic diseases | | | | At least one chronic disease | | | |
| --- | --- | --- | --- | --- | --- | --- | --- | --- |
|  | Aged 50-65 | | Aged 66+ | | Aged 50-65 | | Aged 65+ | |
|  | (1) | (2) | (3) | (4) | (5) | (6) | (7) | (8) |
|  | OLS | FE | OLS | FE | OLS | FE | OLS | FE |
| CPI | -0.0381* | -0.0366 | -0.0958*** | -0.0923*** | -0.0129 | -0.0139 | -0.0233*** | -0.0236*** |
|  | (0.0226) | (0.0226) | (0.0334) | (0.0339) | (0.0101) | (0.0088) | (0.0067) | (0.0073) |
|  |  |  |  |  |  |  |  |  |
| Age | 0.0261*** | 0.0114 | 0.019*** | 0.034 | 0.014*** | -0.0047 | 0.0052*** | 0.0267** |
|  | (0.0013) | (0.0201) | (0.0014) | (0.0366) | (0.0005) | (0.0089) | (0.0004) | (0.0105) |
|  |  |  |  |  |  |  |  |  |
| Female | -0.0937*** | - | -0.0045 | - | -0.0293*** | - | -0.0616 | - |
|  | (0.0137) |  | (0.0192) |  | (0.0051) |  | (0.0073) |  |
|  |  |  |  |  |  |  |  |  |
| Region FE | Yes | No | Yes | No | Yes | No | Yes | No |
| Wave FE | Yes | Yes | Yes | Yes | Yes | Yes | Yes | Yes |
| Socio-Demographics | Yes | Yes | Yes | Yes | Yes | Yes | Yes | Yes |
| Healthy Lifestyle | Yes | Yes | Yes | Yes | Yes | Yes | Yes | Yes |
|  |  |  |  |  |  |  |  |  |
| Mean | 0.864 | 0.864 | 1.497 | 1.497 | 0.527 | 0.527 | 0.75 | 0.75 |
| Observations | 81,173 | 81,173 | 80,468 | 80,468 | 81,173 | 81,173 | 80,468 | 80,468 |
| R-squared | 0.157 | 0.074 | 0.097 | 0.031 | 0.11 | 0.027 | 0.124 | 0.012 |
| Notes. Europe, data from 2004 to 2015. OLS = Ordinary Least Squares; FE = Fixed Effects; CPI = Corruption Perception Index. Pooled OLS (columns 1-3-5-7) and FE (columns 2-4-6-8) estimates of specification (1), using the number and the probability of diseases as outcome variables. The sample is split by age.  Robust standard errors clustered at the regional level in parenthesis. *** p < 0.01, ** p < 0.05, * p < 0.1. | | | | | | | | |

Table A10. The impact of Corruption Perception Index on the number and probability of chronic diseases, sample split by gender.

| *Dependent Variable:* | No. of chronic diseases | | | | At least one chronic disease | | | |
| --- | --- | --- | --- | --- | --- | --- | --- | --- |
|  | Male | | Female | | Male | | Female | |
|  | (1) | (2) | (3) | (4) | (5) | (6) | (7) | (8) |
|  | OLS | FE | OLS | FE | OLS | FE | OLS | FE |
| CPI | -0.0588* | -0.058** | -0.0777*** | -0.078*** | -0.0168* | -0.0178** | -0.0188*** | -0.0213*** |
|  | (0.0305) | (0.0287) | (0.0246) | (0.0241) | (0.009) | (0.0082) | (0.0067) | (0.0068) |
|  |  |  |  |  |  |  |  |  |
| Age | 0.0215*** | 0.0308 | 0.0289*** | 0.0204 | 0.0078*** | 0.0168 | 0.0099*** | 0.007 |
|  | (0.0011) | (0.0321) | (0.0009) | (0.0237) | (0.0004) | (0.011) | (0.0003) | (0.0088) |
|  |  |  |  |  |  |  |  |  |
| Region FE | Yes | No | Yes | No | Yes | No | Yes | No |
| Wave FE | Yes | Yes | Yes | Yes | Yes | Yes | Yes | Yes |
| Socio-Demographics | Yes | Yes | Yes | Yes | Yes | Yes | Yes | Yes |
| Healthy Lifestyle | Yes | Yes | Yes | Yes | Yes | Yes | Yes | Yes |
|  |  |  |  |  |  |  |  |  |
| Mean | 1.191 | 1.191 | 1.169 | 1.169 | 0.646 | 0.646 | 0.632 | 0.632 |
| Observations | 72,182 | 72,182 | 89,459 | 89,459 | 72,182 | 72,182 | 89,459 | 89,459 |
| R-squared | 0.161 | 0.098 | 0.192 | 0.127 | 0.117 | 0.072 | 0.145 | 0.096 |
| Notes. Europe, data from 2004 to 2015. OLS = Ordinary Least Squares; FE = Fixed Effects; CPI = Corruption Perception Index. Pooled OLS (columns 1-3-5-7) and FE (columns 2-4-6-8) estimates of specification (1), using the number and the probability of diseases as outcome variables. The sample is split by gender.  Robust standard errors clustered at the regional level in parenthesis. *** p < 0.01, ** p < 0.05, * p < 0.1. | | | | | | | | |

Table A11. The impact of Corruption Perception Index on the number and probability of chronic diseases, sample split by years of education.

| *Dependent Variable:* | No. of chronic diseases | | | | At least one disease | | | |
| --- | --- | --- | --- | --- | --- | --- | --- | --- |
|  | Low Education | | High Education | | Low Education | | High Education | |
|  | (1) | (2) | (3) | (4) | (5) | (6) | (7) | (8) |
|  | OLS | FE | OLS | FE | OLS | FE | OLS | FE |
| CPI | -0.0789*** | -0.0836*** | -0.0562** | -0.0463 | -0.0195** | -0.0268*** | -0.0176** | -0.0127 |
|  | (0.0301) | (0.0264) | (0.026) | (0.0293) | (0.0081) | (0.007) | (0.0083) | (0.0095) |
|  |  |  |  |  |  |  |  |  |
| Age | 0.0242*** | 0.00766 | 0.029*** | 0.0481** | 0.008*** | 0.0154 | 0.0109*** | 0.011 |
|  | (0.0011) | (0.0315) | (0.001) | (0.0236) | (0.0004) | (0.0101) | (0.0004) | (0.0119) |
|  |  |  |  |  |  |  |  |  |
| Female | -0.0157 | - | -0.0924*** | - | -0.00234 | - | -0.0331*** | - |
|  | (0.0158) |  | (0.018) |  | (0.0059) |  | (0.0062) |  |
|  |  |  |  |  |  |  |  |  |
| Region FE | Yes | No | Yes | No | Yes | No | Yes | No |
| Wave FE | Yes | Yes | Yes | Yes | Yes | Yes | Yes | Yes |
| Socio-Demographics | Yes | Yes | Yes | Yes | Yes | Yes | Yes | Yes |
| Healthy Lifestyle | Yes | Yes | Yes | Yes | Yes | Yes | Yes | Yes |
|  |  |  |  |  |  |  |  |  |
| Mean | 1.315 | 1.315 | 1.009 | 1.009 | 0.683 | 0.683 | 0.583 | 0.583 |
| Observations | 72,182 | 72,182 | 89,459 | 89,459 | 72,182 | 72,182 | 89,459 | 89,459 |
| R-squared | 0.154 | 0.053 | 0.182 | 0.102 | 0.117 | 0.072 | 0.145 | 0.096 |
| Notes. Europe, data from 2004 to 2015. OLS = Ordinary Least Squares; FE = Fixed Effects; CPI = Corruption Perception Index. Pooled OLS (columns 1-3-5-7) and FE (columns 2-4-6-8) estimates of specification (1), using the number and the probability of diseases as outcome variables. The sample is split by years of education.  Robust standard errors clustered at the regional level in parenthesis. *** p < 0.01, ** p < 0.05, * p < 0.1. | | | | | | | | |

Table A12. Heterogeneous effect of Control of Corruption. Individual characteristics.

| *Dependent Variable:* |  |  |  |  |
| --- | --- | --- | --- | --- |
| Number of chronic diseases | OLS | FE | Mean | Obs. |
| Low HH income | -0.141*** | -0.192*** | 1.32 | 80,883 |
|  | (0.0528) | (0.056) |  |  |
| High HH income | -0.122* | -0.133** | 1.038 | 80,758 |
|  | (0.0667) | (0.056) |  |  |
| Aged 50-65 | -0.0944** | -0.138*** | 0.864 | 81,173 |
|  | (0.0443) | (0.0408) |  |  |
| Aged 66+ | -0.175** | -0.21*** | 1.497 | 80,468 |
|  | (0.0709) | (0.0726) |  |  |
| Male | -0.16** | -0.198*** | 1.191 | 72,182 |
|  | (0.0723) | (0.0569) |  |  |
| Female | -0.126*** | -0.168*** | 1.169 | 89,459 |
|  | (0.0474) | (0.0506) |  |  |
| Low Education | -0.151** | -0.213*** | 1.315 | 89,638 |
|  | (0.063) | (0.0563) |  |  |
| High Education | -0.104* | -0.114** | 1.009 | 72,003 |
|  | (0.0558) | (0.0468) |  |  |
| Notes. Europe, data from 2004 to 2015. OLS = Ordinary Least Squares; FE = Fixed Effects; HH = Household. Pooled OLS (column 1) and FE (column 2) estimates of specification (1).Socio-demographics include years of education, household income, job status, marital status, place of living, and the number of children. Healthy lifestyle include smoking behaviour, alcohol consumption, sport engagement, and the Body Mass Index category. The sample is split by gender, median household income, age, and years of education.  Robust standard errors clustered at the regional level in parenthesis. *** p < 0.01, ** p < 0.05, * p < 0.1. | | | | |

Table A13. The impact of sectorial corruption on the number of chronic diseases.

| Sector | $PC_{s}$ | St. Dev. | Obs. | $R^{2}$ |
| --- | --- | --- | --- | --- |
| *Pooled OLS* |  |  |  |  |
| Healthcare | 0.00407*** | (0.00121) | 149,119 | 0.177 |
| Police | 0.0004 | (0.0008) | 149,119 | 0.177 |
| Judicial | 0.00440** | (0.00188) | 149,119 | 0.177 |
| Education | 0.00222 | (0.00162) | 149,119 | 0.177 |
| Tenders | 0.000708 | (0.00147) | 149,119 | 0.177 |
| Politicians | 0.00258*** | (0.000836) | 149,119 | 0.177 |
| Buildings | -0.000976 | (0.00140) | 149,119 | 0.177 |
| Business | 0.000503 | (0.00122) | 149,119 | 0.177 |
|  |  |  |  |  |
| *Panel FE* |  |  |  |  |
| Healthcare | 0.00468*** | (0.00128) | 149,119 | 0.016 |
| Police | 0.0006 | (0.0008) | 149,119 | 0.015 |
| Judicial | 0.00387* | (0.00196) | 149,119 | 0.016 |
| Education | 0.00237 | (0.00152) | 149,119 | 0.015 |
| Tenders | 0.00219 | (0.00134) | 149,119 | 0.015 |
| Politicians | 0.00294*** | (0.000912) | 149,119 | 0.016 |
| Buildings | 0.000865 | (0.00121) | 149,119 | 0.015 |
| Business | 0.00203* | (0.00108) | 149,119 | 0.015 |
| Notes. Europe, data from 2004 to 2015. PC_s_ = Perceived Corruption in sector s as in column 1; OLS = Ordinary Least Squares; St. Dev. = Standard Deviation; Obs. = Observations. Pooled OLS and FE estimates of specification (1), with PC_s_ for each sector s as a health outcome variable. All estimates include region and wave fixed-effects, socio-demographics (age, gender, years of education, household income, job status,  marital status, place of living, and the number of children), and healthy lifestyle (smoking behaviour, alcohol consumption, sport engagement, and the Body Mass Index category).  Robust standard errors clustered at the regional level in parenthesis. *** p < 0.01, ** p < 0.05, * p < 0.1. | | | | |

**Appendix B. Empirical Methodologies**

**Pooled Ordinary Least Squares (OLS) and Fixed-Effects Panel Model**

We employ two different statistical methodologies to explore the relationship between corruption and individual healthcare indicators, namely pooled ordinary least squares (OLS) and fixed-effects panel regression. OLS (respectively, panel models) assume a linear relationship between the dependent variable *y_i_* (*y_it_*) and a vector of regressors **x***_i_* (**x***_it_*) for each observed individual *i* (and each period *t*), plus a random error term *ε_i_* (*ε_it_*). Both methodologies obtain the estimators of regressors by minimising the sum of squared residuals (Wooldridge, 2002; Green, 2003). The main difference between the two regression models lies in the different assumptions concerning the serial correlation between observations belonging to the same individual (panels). In particular, while pooled OLS treats all observations in the sample as independent among each other, panel models account for the within-panel correlation between observations referring to the same statistical unit (in our case, an individual). As a consequence, pooled OLS estimates are potentially biased in cases of within-panel correlation.

Fixed-effects models are panel models which assume correlation between unobserved individual heterogeneity and observed variables, and therefore group-specific means do not vary over time. Alternatively, random-effects models are panel models that consider unobserved individual heterogeneity being uncorrelated with observed variables and an individual random component affecting group-specific means. The choice of a fixed-effects rather than a random-effects model is justified on the basis of an overidentification test on the additional orthogonality restrictions implied by random-effects (Wooldridge, 2002). The results of this test are significant at the 1% confidence level, and confirm that these restrictions overidentify the model (Sargan-Hansen statistic = 2,635. Chi-squared(25), P-value = 0. As a consequence, we are precluded from the use of the random-effects estimator.

Panel data methodologies, moreover, are successful in solving the bias caused by omitted individual time-invariant characteristics. If individuals are observed for more than one time period, in fact, it is possible to estimate a model in the differences of all the variables and thus get rid of these unobserved time-invariant characteristics. Panel estimation, however, does not tackle any endogeneity issues stemming from time-varying unobserved factors. Our results, as a consequence, are better expressed in terms of correlation than of causality.

**Data**

*SHARE*

The consistent use of panel methodologies requires, as pointed out in the previous section, the same statistical unit to be observed repeatedly over time. SHARE generally satisfies this requirement, as most respondents are surveyed in more than one wave. However, some countries (i.e., Croatia, Hungary, Ireland, and Portugal) participated in one wave only and therefore individuals in these countries are not observed over time. In a similar fashion, there are instances of individuals who were surveyed in one wave only even if their country of residence participated in more than one wave. In order to consistently apply panel analysis, we thus exclude individuals that were surveyed in one wave only. The results of our analysis are not affected by this restriction.

Waves 1, 2, and 4 in SHARE were run over different years. In particular, wave 1 was carried out in the years 2004-2005, wave 2 in 2006-2007, and wave 4 in 2010-2012. In order for a yearly measure of perceived corruption to be consistently employed in the analysis, however, we have considered the level of CPI corresponding to the year of the interview reported by each respondent in that particular wave. This information is available for all respondents in SHARE.

In our econometric specification, we include wave dummies to control for any unobserved time trends that could affect the individual number of chronic diseases. Results are not qualitatively affected if we replace wave fixed effects with year-of-interview fixed effects. The small amount of observations characterising some of the years in our sample (especially 2005, 2010, and 2012), however, negatively affects the precision of estimates.

Moreover, we include regional dummies at the NUTS 2 (Nomenclature des unités territoriales statistiques) level to control for any unobserved geographic characteristics that could affect our morbidity indicator. While SHARE provides the region of residence at NUTS 2 level for most of the individuals surveyed in our sample, this information is missing for respondents from Germany and for the Netherlands (waves 4 and 5). In cases where the region of residence at NUTS 2 level is missing, we resort to NUTS 1 classification, which is available for all waves and countries. Results are not qualitatively affected if we include NUTS 1 dummies as regional fixed effects.

In wave 6 question on alcohol consumption asks to report the frequency of consumption only if this exceeds six drinks. Therefore, responses in waves 1–5 and in wave 6 are not comparable. In order to maximise our sample, we attach alcohol consumption in wave 5 to respondents who are also surveyed in wave 6. To check the consistency of this choice, we run our baseline specification on a sample including all waves but 6. Estimates show that this exclusion does not qualitatively affect our analysis; on the contrary, the coefficient increases and this proves that the bias from the inclusion of observations in wave 6 is negative, if any.

In the paper, we run our baseline regression separately on different categories of chronic diseases. The latter are grouped according to the apparatus they primarily affect. Cardiovascular diseases include diabetes, heart attack, high blood pressure or hypertension, high cholesterol, and stroke. Age diseases account for Parkinson disease and cataracts. Finally, respiratory diseases include chronic lung disease only. To ensure comparability with our main analysis, we exclude morbidities that SHARE does not include in the overall number of chronic diseases, as they are not present in all waves. These are Alzheimer, asthma, arthritis, emotional disorders, and osteoporosis.

**Eurobarobeter**

We use data on corruption provided in Eurobarometer 64.3, 68.2, 72.2, 76.1, 79.1, and 88.2. Eurobarometer surveys report individual multiple choice answers to the questions: “In [your country], do you think that the giving and taking of bribes and the abuse of power for personal gain are widespread among any of the following [sector]?” for the years 2005, 2007, 2009, 2011, 2013, and 2017.

Since Eurobarometer is recorded every two years from 2005 to 2013, and in 2017, we link each year of Eurobarometer survey with the same year and the following year of SHARE survey. The year 2015 is the average between 2013 and 2017 measures. Results do not significantly change if we consider a moving average between consecutive years or the observed years only.

**Sample split**

Countries with GDP per capita higher than the median are Austria, Belgium, Denmark, France, Germany, Italy, Netherlands, Spain (for the years 2013-2015), and Sweden; countries with lower median GDP are Czech Republic, Estonia, Greece, Poland, Slovenia, and Spain (for the years 2004-2012); countries with higher median health expenditure per capita are Austria, Belgium, Denmark, France, Germany, Italy, Netherlands, and Sweden; countries with lower median health expenditure are Czech Republic, Estonia, Greece, Italy (in 2015 only), Poland, Slovenia, and Spain.

**References**

Greene WH (2003) Econometric analysis. Pearson Education India.

Wooldridge JM (2010) Econometric analysis of cross section and panel data. MIT press.
